# Supplementary material for: The Impact of Severe Maternal Morbidity on Perinatal Outcomes in High Income Countries: Systematic Review and Meta-Analysis
Source: J Clin Med. 2020 Jun 29;9(7):2035. doi: 10.3390/jcm9072035 (PMC7409239; doi:10.3390/jcm9072035)
Supplement: Supplementary file 1 [file jcm-09-02035-s001.zip › supplement files/Supplementary File 1_Search Strategy_PubMed.docx]

**Supplementary files**

eFile 1; Sample search strategy for PubMed database

((((((((((((("peripartum cardiomyopathy"[Title/Abstract] OR shock[Title/Abstract] OR "cardiac arrest"[Title/Abstract] OR arrhythmia[Title/Abstract] OR "cardiopulmonary resuscitation"[Title/Abstract] OR "severe hypoperfusion"[Title/Abstract]) OR ("severe acidosis"[Title/Abstract] OR "acute cyanosis"[Title/Abstract] OR gasping[Title/Abstract] OR intubation[Title/Abstract] OR ventilation[Title/Abstract])) OR ("severe hypoxemia"[Title/Abstract] OR "oliguria"[Title/Abstract] OR "acute renal failure"[Title/Abstract] OR "acute kidney injury"[Title/Abstract] OR dialysis[Title/Abstract])) OR ("amniotic fluid embolism"[Title/Abstract] OR "pulmonary embolism"[Title/Abstract] OR "pulmonary edema"[Title/Abstract] OR "deep vein thrombosis"[Title/Abstract] OR clotting[Title/Abstract] OR coagulopathy[Title/Abstract])) OR ("severe acute thrombocytopenia"[Title/Abstract] OR "acute fatty liver"[Title/Abstract] OR "severe liver injury"[Title/Abstract] OR cholecystitis[Title/Abstract] OR "intrahepatic cholestasis of pregnancy"[Title/Abstract] OR "liver failure"[Title/Abstract])) OR (coma[Title/Abstract] OR seizure[Title/Abstract] OR stroke[Title/Abstract] OR "transient ischemic attack"[Title/Abstract] OR "ischaemic stroke"[Title/Abstract])) OR ("status epilepticus"[Title/Abstract] OR "acute epileptic seizure"[Title/Abstract] OR "cerebrovascular accident"[Title/Abstract] OR paralysis[Title/Abstract])) OR ("maternal near miss"[Title/Abstract] OR "obstetric near miss"[Title/Abstract] OR "near miss morbidity"[Title/Abstract] OR "obstetric near-miss"[Title/Abstract] OR hysterectomy[Title/Abstract])) OR ("uterine rupture"[Title/Abstract] OR "emergency obstetric hysterectomy"[Title/Abstract] OR "maternal complications"[Title/Abstract] OR "severe maternal morbidity"[Title/Abstract])) OR ("severe acute maternal morbidity"[Title/Abstract] OR "pregnancy complications"[Title/Abstract] OR "intensive care unit admission"[Title/Abstract] OR "blood transfusion"[Title/Abstract])) OR ("obstetric hemorrhage"[Title/Abstract] OR "severe postpartum haemorrhage"[Title/Abstract] OR haemorrhage[Title/Abstract] OR "severe pre-eclampsia"[Title/Abstract] OR eclampsia[Title/Abstract] OR "HELLP syndrome"[Title/Abstract] OR "severe preeclampsia"[Title/Abstract] OR "severe gestational hypertension"[Title/Abstract])) OR (chorioamnionitis[Title/Abstract] OR sepsis[Title/Abstract] OR endometritis[Title/Abstract] OR "renal failure"[Title/Abstract])) AND ((((("perinatal morbidity"[Title/Abstract] OR "adverse outcome"[Title/Abstract] OR "neonatal mortality"[Title/Abstract] OR "neonatal morbidity"[Title/Abstract] OR "neonatal death"[Title/Abstract]) OR (stillbirth[Title/Abstract] OR "fetal death"[Title/Abstract] OR "perinatal death"[Title/Abstract] OR "perinatal mortality"[Title/Abstract] OR "small for gestational age"[Title/Abstract])) OR ("low birth weight"[Title/Abstract] OR "preterm birth"[Title/Abstract] OR "Apgar score"[Title/Abstract] OR "neonatal acidosis"[Title/Abstract] OR "NICU admission"[Title/Abstract])) OR ("neonatal intensive care admission"[Title/Abstract] OR "hypoxic-ischemic encephalopathy"[Title/Abstract] OR "periventricular leukomalacia"[Title/Abstract] OR "interventricular haemorrhage"[Title/Abstract])) OR (("preterm birth"[Title/Abstract] OR "preterm infant"[Title/Abstract]) OR ("premature birth"[Title/Abstract] OR "premature infants"[Title/Abstract])))) AND (((((((((((((((((((((((((((((((((((((((((((((((((((((((((((((((((((((((((((((((((("andorra"[MeSH Terms] OR "andorra"[All Fields]) OR ("antigua and barbuda"[MeSH Terms] OR ("antigua"[All Fields] AND "barbuda"[All Fields]) OR "antigua and barbuda"[All Fields])) OR ("aruba"[MeSH Terms] OR "aruba"[All Fields])) OR ("australia"[MeSH Terms] OR "australia"[All Fields])) OR ("austria"[MeSH Terms] OR "austria"[All Fields])) OR ("bahamas"[MeSH Terms] OR "bahamas"[All Fields])) OR ("bahrain"[MeSH Terms] OR "bahrain"[All Fields])) OR ("barbados"[MeSH Terms] OR "barbados"[All Fields])) OR ("belgium"[MeSH Terms] OR "belgium"[All Fields])) OR ("bermuda"[MeSH Terms] OR "bermuda"[All Fields])) OR ("british virgin islands"[MeSH Terms] OR ("british"[All Fields] AND "virgin"[All Fields] AND "islands"[All Fields]) OR "british virgin islands"[All Fields])) OR ("brunei"[MeSH Terms] OR "brunei"[All Fields] OR ("brunei"[All Fields] AND "darussalam"[All Fields]) OR "brunei darussalam"[All Fields])) OR ("canada"[MeSH Terms] OR "canada"[All Fields])) OR ("west indies"[MeSH Terms] OR ("west"[All Fields] AND "indies"[All Fields]) OR "west indies"[All Fields] OR ("cayman"[All Fields] AND "islands"[All Fields]) OR "cayman islands"[All Fields])) OR ("channel islands"[MeSH Terms] OR ("channel"[All Fields] AND "islands"[All Fields]) OR "channel islands"[All Fields])) OR ("chile"[MeSH Terms] OR "chile"[All Fields])) OR ("croatia"[MeSH Terms] OR "croatia"[All Fields])) OR ("curacao"[MeSH Terms] OR "curacao"[All Fields])) OR ("cyprus"[MeSH Terms] OR "cyprus"[All Fields])) OR ("czech republic"[MeSH Terms] OR ("czech"[All Fields] AND "republic"[All Fields]) OR "czech republic"[All Fields])) OR ("denmark"[MeSH Terms] OR "denmark"[All Fields])) OR ("estonia"[MeSH Terms] OR "estonia"[All Fields])) OR ("france"[MeSH Terms] OR "france"[All Fields])) OR ("polynesia"[MeSH Terms] OR "polynesia"[All Fields] OR ("french"[All Fields] AND "polynesia"[All Fields]) OR "french polynesia"[All Fields])) OR (("denmark"[MeSH Terms] OR "denmark"[All Fields] OR ("faroe"[All Fields] AND "islands"[All Fields]) OR "faroe islands"[All Fields]) AND ("finland"[MeSH Terms] OR "finland"[All Fields]))) OR ("germany"[MeSH Terms] OR "germany"[All Fields])) OR ("gibraltar"[MeSH Terms] OR "gibraltar"[All Fields])) OR ("greece"[MeSH Terms] OR "greece"[All Fields])) OR ("greenland"[MeSH Terms] OR "greenland"[All Fields])) OR ("guam"[MeSH Terms] OR "guam"[All Fields])) OR ("hong kong"[MeSH Terms] OR ("hong"[All Fields] AND "kong"[All Fields]) OR "hong kong"[All Fields])) OR ("hungary"[MeSH Terms] OR "hungary"[All Fields])) OR ("iceland"[MeSH Terms] OR "iceland"[All Fields])) OR ("ireland"[MeSH Terms] OR "ireland"[All Fields])) OR ("united kingdom"[MeSH Terms] OR ("united"[All Fields] AND "kingdom"[All Fields]) OR "united kingdom"[All Fields] OR ("isle"[All Fields] AND "man"[All Fields]) OR "isle of man"[All Fields])) OR ("israel"[MeSH Terms] OR "israel"[All Fields])) OR ("italy"[MeSH Terms] OR "italy"[All Fields])) OR ("japan"[MeSH Terms] OR "japan"[All Fields])) OR ("korea"[MeSH Terms] OR "korea"[All Fields])) OR ("kuwait"[MeSH Terms] OR "kuwait"[All Fields])) OR ("latvia"[MeSH Terms] OR "latvia"[All Fields])) OR ("liechtenstein"[MeSH Terms] OR "liechtenstein"[All Fields])) OR ("lithuania"[MeSH Terms] OR "lithuania"[All Fields])) OR ("luxembourg"[MeSH Terms] OR "luxembourg"[All Fields])) OR (("macau"[MeSH Terms] OR "macau"[All Fields]) AND SAR[All Fields])) OR ("malta"[MeSH Terms] OR "malta"[All Fields])) OR ("monaco"[MeSH Terms] OR "monaco"[All Fields])) OR ("micronesia"[MeSH Terms] OR "micronesia"[All Fields] OR "nauru"[All Fields])) OR ("netherlands"[MeSH Terms] OR "netherlands"[All Fields])) OR ("new caledonia"[MeSH Terms] OR ("new"[All Fields] AND "caledonia"[All Fields]) OR "new caledonia"[All Fields])) OR ("new zealand"[MeSH Terms] OR ("new"[All Fields] AND "zealand"[All Fields]) OR "new zealand"[All Fields])) OR ("micronesia"[MeSH Terms] OR "micronesia"[All Fields] OR ("northern"[All Fields] AND "mariana"[All Fields] AND "islands"[All Fields]) OR "northern mariana islands"[All Fields])) OR ("norway"[MeSH Terms] OR "norway"[All Fields])) OR ("oman"[MeSH Terms] OR "oman"[All Fields])) OR (("poland"[MeSH Terms] OR "poland"[All Fields]) AND ("portugal"[MeSH Terms] OR "portugal"[All Fields]))) OR ("puerto rico"[MeSH Terms] OR ("puerto"[All Fields] AND "rico"[All Fields]) OR "puerto rico"[All Fields])) OR ("qatar"[MeSH Terms] OR "qatar"[All Fields])) OR ("san marino"[MeSH Terms] OR ("san"[All Fields] AND "marino"[All Fields]) OR "san marino"[All Fields])) OR ("saudi arabia"[MeSH Terms] OR ("saudi"[All Fields] AND "arabia"[All Fields]) OR "saudi arabia"[All Fields])) OR ("seychelles"[MeSH Terms] OR "seychelles"[All Fields])) OR ("singapore"[MeSH Terms] OR "singapore"[All Fields])) OR ("sint maarten"[MeSH Terms] OR ("sint"[All Fields] AND "maarten"[All Fields]) OR "sint maarten"[All Fields])) OR ("slovakia"[MeSH Terms] OR "slovakia"[All Fields] OR ("slovak"[All Fields] AND "republic"[All Fields]) OR "slovak republic"[All Fields])) OR ("slovenia"[MeSH Terms] OR "slovenia"[All Fields])) OR ("spain"[MeSH Terms] OR "spain"[All Fields])) OR ("saint kitts and nevis"[MeSH Terms] OR ("saint"[All Fields] AND "kitts"[All Fields] AND "nevis"[All Fields]) OR "saint kitts and nevis"[All Fields] OR ("st"[All Fields] AND "kitts"[All Fields] AND "nevis"[All Fields]) OR "st kitts and nevis"[All Fields])) OR (Martin, St[Full Author Name] OR (Martin, St[Full Author Name] OR St. Martin[Author] OR St. Martin[Investigator]) OR St. Martin[Investigator])) OR ("sweden"[MeSH Terms] OR "sweden"[All Fields])) OR ("switzerland"[MeSH Terms] OR "switzerland"[All Fields])) OR ("taiwan"[MeSH Terms] OR "taiwan"[All Fields])) OR ("trinidad and tobago"[MeSH Terms] OR ("trinidad"[All Fields] AND "tobago"[All Fields]) OR "trinidad and tobago"[All Fields])) OR ("west indies"[MeSH Terms] OR ("west"[All Fields] AND "indies"[All Fields]) OR "west indies"[All Fields] OR ("turks"[All Fields] AND "caicos"[All Fields] AND "islands"[All Fields]) OR "turks and caicos islands"[All Fields])) OR ("united arab emirates"[MeSH Terms] OR ("united"[All Fields] AND "arab"[All Fields] AND "emirates"[All Fields]) OR "united arab emirates"[All Fields])) OR ("united arab emirates"[MeSH Terms] OR ("united"[All Fields] AND "arab"[All Fields] AND "emirates"[All Fields]) OR "united arab emirates"[All Fields])) OR ("united kingdom"[MeSH Terms] OR ("united"[All Fields] AND "kingdom"[All Fields]) OR "united kingdom"[All Fields])) OR ("united states"[MeSH Terms] OR ("united"[All Fields] AND "states"[All Fields]) OR "united states"[All Fields])) OR ("uruguay"[MeSH Terms] OR "uruguay"[All Fields])) OR ("united states virgin islands"[MeSH Terms] OR ("united"[All Fields] AND "states"[All Fields] AND "virgin"[All Fields] AND "islands"[All Fields]) OR "united states virgin islands"[All Fields] OR ("virgin"[All Fields] AND "islands"[All Fields]) OR "virgin islands"[All Fields])) OR UK[All Fields]) OR UAE[All Fields]) OR USA[All Fields]) OR US[All Fields]) AND (Journal Article[ptyp] AND "loattrfull text"[sb] AND "humans"[MeSH Terms] AND English[lang])
