# Supplementary material for: The Impact of Severe Maternal Morbidity on Perinatal Outcomes in High Income Countries: Systematic Review and Meta-Analysis
Source: J Clin Med. 2020 Jun 29;9(7):2035. doi: 10.3390/jcm9072035 (PMC7409239; doi:10.3390/jcm9072035)
Supplement: Supplementary file 1 [file jcm-09-02035-s001.zip › supplement files/Supplementary File 2_Funnel plots for publication bias.docx]

Supplementary File 2


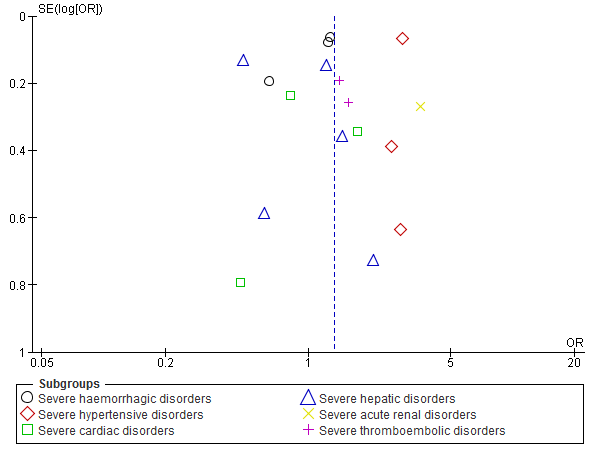

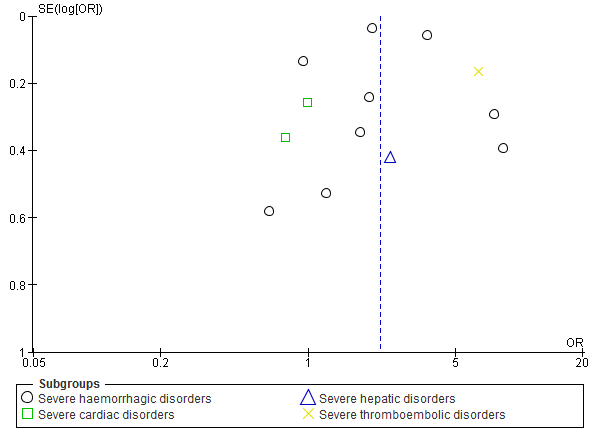

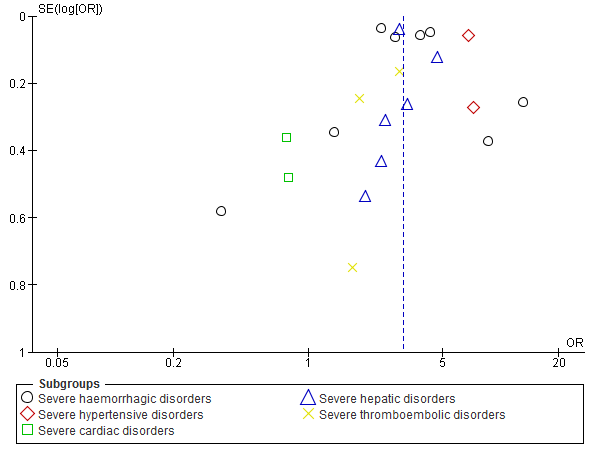


A

C

B


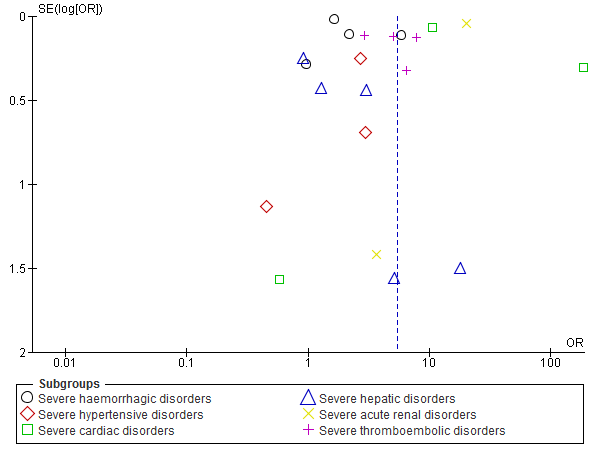

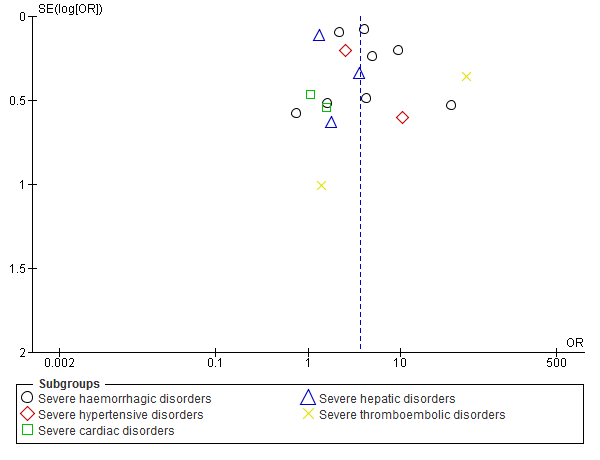

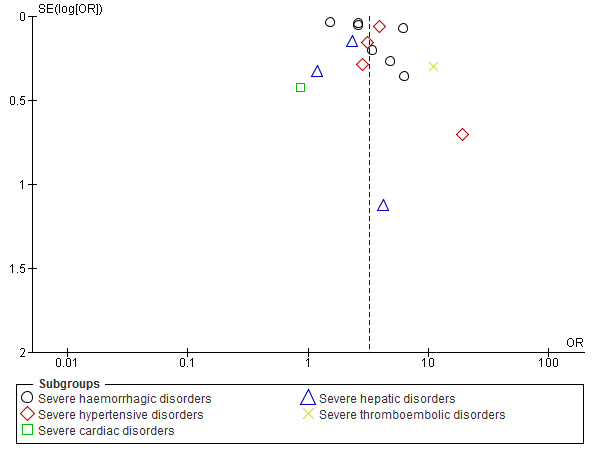


D

F

E


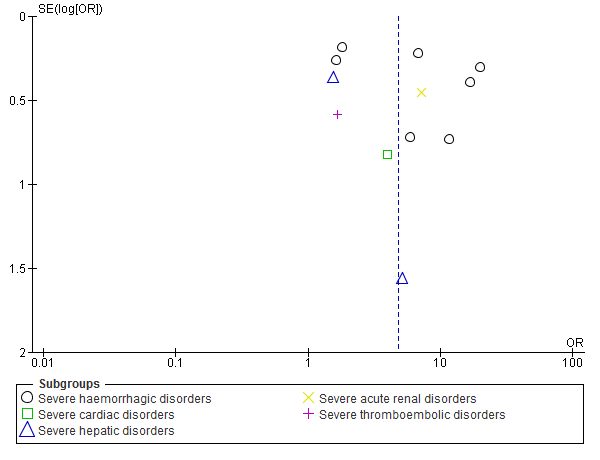

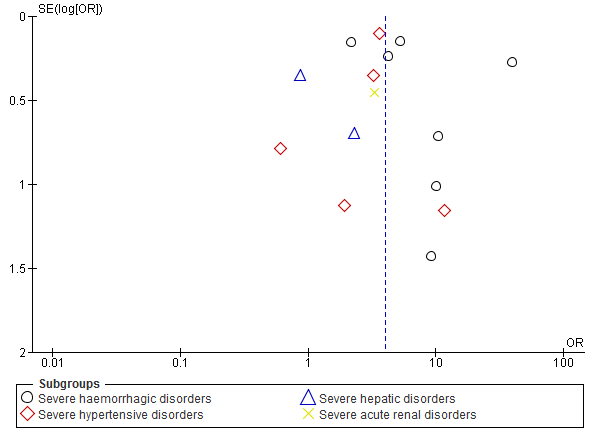


G

H

Supplementary Figure 1. Funnel plot of publication bias A. preterm birth, B. small for gestational age, C. low birth weight (2500g), D. 5-minute Apgar score (<7) E. admission to NICU, F. stillbirth/fetal death, G. neonatal death, H. perinatal death
